# Effects of occupational factors on depression in Chinese veterans: a fsQCA study based on 2022 CFPS data

**DOI:** 10.3389/fpsyg.2026.1853613

**Published:** 2026-06-19

**Authors:** Lina Zhao, Mengyue Zhang, Zhimin Zhang, Hongyan Guo

**Affiliations:** 1 School of Humanities and Social Sciences, Jiangsu University of Science and Technology, Zhenjiang, China; 2 School of Marxism, Jiangsu University of Science and Technology, Zhenjiang, China

**Keywords:** depression, mental health, occupational factors, occupational health and safety, veterans

## Abstract

**Aim:**

This study examines how multiple occupational attributes, including job satisfaction, occupational prestige, job stability, job decision authority, remuneration, and formal employment status, combine in relation to depressive symptoms among Chinese veterans.

**Methods:**

This study employs fuzzy-set Qualitative Comparative Analysis (fsQCA) to examine configurational associations, which is particularly suitable for outcomes shaped by multiple interacting conditions. Using data from the 2022 China Family Panel Studies (2022 CFPS), we focus on veterans and investigate how combinations of occupational factors are associated with depression, thereby complementing conventional variable-centered linear approaches in occupational health and safety (OHS) research. To improve data quality, we excluded non-veterans, veterans beyond working age, student veterans, and observations with missing values or extreme outliers. The final analytic sample comprises 247 cases.

**Results:**

The findings underscore the asymmetric nature of configurational relationships. No stable sufficient configuration was identified for the high-depression outcome in either the baseline model or robustness checks, suggesting that elevated depression may be shaped by more complex antecedents or by factors beyond the occupational domain. By contrast, 10 configurations were identified for the low-depression outcome, with acceptable overall solution consistency and coverage. Formal employment, job satisfaction, remuneration, occupational prestige, and job stability emerged as key configurational elements associated with low depression. Job decision authority appeared to be context-dependent rather than a core condition for low depression.

**Conclusion:**

Ten distinct configurations associated with low depression were identified. The findings suggest the importance of strengthening formal labor protection, improving person-job fit and job satisfaction, ensuring fair remuneration, enhancing occupational recognition, and supporting employment stability among veterans. Because this study uses cross-sectional data, the configurations should be interpreted as occupational patterns associated with depression outcomes rather than as definitive causal or intervention effects.

## 1 Introduction

Existing studies indicate that veterans are at higher risk of depression than the general population, and depressive symptoms may persist across the life course (Shah and Blais, 2025; Byers et al., 2012; Zhao and Guo, 2022; Goldstein et al., 2025; Fiedler et al., 2006; Tassew et al., 2024; Carlo et al., 2024). Since the founding of the People’s Republic of China, the cumulative number of veterans has reached approximately 57 million, substantially exceeding that of the United States (Shi, 2025). Li et al. (2022) surveyed 2,400 veterans from 12 cities across China’s three major economic regions using the Patient Health Questionnaire-9 (PHQ-9) and a self-developed questionnaire, and reported that 39% screened positive for at least mild depressive symptoms—higher than the 16.3% prevalence reported in the general population. Moreover, 16.8% of veterans met the threshold for clinically significant depressive symptoms, compared with a 6.8% prevalence of depressive disorders in the general population in China.

Depression among veterans constitutes an important public health concern and may impose substantial individual and societal burdens. Existing evidence indicates that depressive symptoms in this population are common and are associated with poorer cognitive functioning and an elevated risk of cognitive decline and dementia, which can lead to functional limitations and reduced quality of life (Byers and Yaffe, 2014). Beyond its clinical consequences, depression is linked to greater healthcare utilization, including more frequent visits to primary and specialty care providers (Ford et al., 2004), and to marked productivity losses; for example, depressed individuals reportedly lose an average of 5.6 working hours per week, compared with 1.6 h among non-depressed workers (Stewart et al., 2003). Moreover, depression-related social withdrawal may further undermine veterans’ wellbeing and increase demands on family caregiving and support systems (Tsai et al., 2009). In China, government policy documents, including the “Opinions on Promoting Employment and Entrepreneurship for Veterans in the New Era” and the “Guiding Opinions on Comprehensively Improving the Education and Training Work for Demobilized Soldiers”, emphasize strengthening psychological services for veterans. The “Work Standards for Assisting and Supporting Veterans in Difficulty” also recommends that veterans’ affairs departments provide psychological counseling through targeted initiatives, project-based activities, and material support. Accordingly, to develop effective prevention and intervention strategies, it is essential to identify the factors that directly and substantially influence depression levels among veterans.

Occupational factors, such as job satisfaction, occupational prestige, job security, decision authority, remuneration, and the presence of formal labor contracts, are central to OHS frameworks for evaluating and mitigating mental health risks, particularly depression among workers (Karasek, 1979; Siegrist, 1996; Tuan and Loan, 2025). Empirical studies indicate that adverse psychosocial work exposures, including low reward, limited decision authority, and insecure employment, are associated with elevated risks of depression and burnout (Belloni et al., 2022; Pandey, 2025; Tran et al., 2025). In addition, the notion of “decent work,” which encompasses fair wages, secure employment, and adequate working conditions, has been linked to reduced stress and improved overall wellbeing (Ghai, 2003; Rantanen et al., 2020). When occupational conditions meet decent work standards, they may help prevent adverse mental health outcomes and enhance job satisfaction, which is itself a robust determinant of mental health (Goetsch and Ozon, 2019). For military veterans, separation from the armed forces often constitutes a major occupational transition (Becker et al., 2023). Existing empirical evidence suggests that many veterans experience reintegration difficulties, and these challenges are closely related to post-service occupational conditions (Romaniuk and Kidd, 2018; Romaniuk et al., 2020; Sayer et al., 2011). Such difficulties during the transition from military to civilian employment have been associated with heightened anxiety and major depressive disorder (Elnitsky et al., 2013; Sayer et al., 2014; Tran et al., 2016). Moreover, suboptimal occupational circumstances may contribute to perceptions of injustice and adversarial attitudes, potentially exacerbating psychological distress (Hu and Tong, 2021).

China faces a pressing need to address depression among its veteran population. Prior research has established links between occupational conditions and depression, yet evidence specific to Chinese veterans remains limited. Existing studies have primarily documented the presence of depressive problems in this group, while providing insufficient insight into the occupational determinants and the mechanisms through which they operate. Moreover, the literature rarely examines how configurations of occupational conditions, rather than isolated factors, shape depression outcomes among veterans. To fill these gaps, this study adopts a configurational approach to investigate how multiple occupational attributes, including job satisfaction, occupational prestige, job stability, job decision authority, remuneration, and formal employment status, jointly relate to depressive symptoms in Chinese veterans. By identifying occupational profiles associated with higher or lower levels of depressive symptoms, we aim to clarify how workplace conditions collectively contribute to veterans’ mental health.

## 2 Materials and methods

### 2.1 Statistical analyses

This study employs fsQCA, a set-theoretic method that links specific configurations of causal conditions to an outcome. In fsQCA, variables are calibrated into fuzzy sets, with membership scores ranging from 0, indicating full non-membership, to 1, indicating full membership, rather than being modeled through linear or additive effects (Ragin and Rihoux, 2004). Unlike conventional regression, which estimates the net effects of individual predictors, fsQCA emphasizes causal complexity and asymmetry. It allows multiple distinct combinations of conditions to produce the same outcome (Vis, 2012). This method is well suited to the current study because it examines how occupational factors jointly relate to depression among Chinese veterans. These relationships are likely to involve complex interactions and context-dependent effects that conventional regression models may overlook. In addition, fsQCA can identify necessary and/or sufficient configurations of conditions, accommodate fuzzy, binary, and multi-valued variables, and handle small-to-medium sample sizes, particularly when higher-order interactions are difficult to interpret using regression-based approaches (Li et al., 2024). Therefore, fsQCA offers a more nuanced and theoretically appropriate approach than conventional regression for studying configurational and asymmetric relationships.

### 2.2 Data sources

This study utilized data from the 2022 CFPS. The CFPS is a nationally representative, large-scale social survey conducted by the Institute of Social Science Survey at Peking University. Key features of the CFPS include: first, it encompasses a large and diverse sample of households and individuals from 25 provinces across China, ensuring that the data is representative of the national population; second, it employs standardized questionnaires, ensuring consistency and comparability of data across different waves and regions. The CFPS is an invaluable tool for understanding the complex dynamics of Chinese society, offering insights crucial for informed policymaking and academic research. The dataset includes information on veterans’ depression and occupational factors. To minimize the impact of data noise on the empirical results, we excluded non-veteran samples, veterans exceeding the working age (for males: 16–60 years old; for females: 16–55 years old), veterans who are students, and those with missing variables or outliers. The original veteran sample comprised 741 individuals; 494 invalid cases were excluded (66.67%), and 247 valid cases were retained (33.33%).

The sample was predominantly male, with 242 male veterans (98.0%) and 5 female veterans (2.0%), reflecting the male-dominated composition of the veteran population. Regarding household registration, 110 veterans (44.7%) had agricultural hukou, 86 (35.0%) had non-agricultural hukou, 50 (20.3%) had resident hukou and 1 veteran reported not knowing their household registration type. In terms of marital status, 190 veterans (76.9%) were married or cohabiting, 37 (15.0%) were never married, 18 (7.3%) were divorced, 2 (0.8%) were widowed.

To assess potential selection bias, we further compared the final analytic sample with the excluded cases from the original veteran dataset. The original dataset contained 741 veteran cases; after excluding invalid cases and cases that did not meet the age and employment criteria, 247 cases were retained and 494 cases were excluded. Because age range and employment status were used as eligibility criteria, they were not included in the comparison. Instead, we compared the included and excluded cases on sex, household registration type, and marital status. The results showed no significant difference in sex composition between the two groups (*p* = 0.618). However, significant differences were observed in household registration type (*p* < 0.001) and marital status (*p* < 0.001). Compared with excluded cases, the included cases had a lower proportion of agricultural hukou and a higher proportion of non-agricultural hukou. The included cases also had a higher proportion of never-married and divorced veterans and a lower proportion of widowed veterans. These findings suggest that potential selection bias cannot be fully ruled out. Therefore, the results should be interpreted as applying primarily to currently employed, working-age veterans with complete information on the variables used in this study, rather than to the entire veteran population.

Regarding sample size adequacy, the final analytic sample included 247 veterans and six antecedent conditions. These six conditions theoretically generate 2^6^ = 64 possible truth-table configurations. In the baseline fsQCA analysis, a frequency cutoff of 2 was used to reduce reliance on single-case configurations. In addition, robustness checks using alternative threshold specifications showed substantively stable results. Thus, the sample size was considered acceptable for the exploratory configurational analysis conducted in this study. Nevertheless, future studies with larger veteran samples are needed to further validate the identified configurations.

### 2.3 Variables definition

Veterans’ depression. The Center for Epidemiologic Studies Depression Scale (CES-D) is a widely used, freely available self-report measure for assessing depressive symptoms (Carleton et al., 2013). In this study, the variables related to veterans’ depression are based on the CES-D 8-item version. The CES-D8 consists of 8 items: Did you feel depressed? Did you feel that everything you did was an effort? Was your sleep restless? Were you happy? Did you feel lonely? Did you enjoy life? Did you feel sad? Were you unable to get going? Responses for each item range from 0 (none or almost none of the time) to 3 (all or almost all of the time) (de Van Vel et al., 2009). The CES-D 8 scale has been widely used in studies to assess depression in the general population, and its validity has been established (Missinne et al., 2014). In the 2022 CFPS, the CES-D8 (an abbreviated version of the CES-D 20) was used to measure depressive symptoms. This decision was based on feedback from field interviewers, who reported that the 20-item CES-D was less acceptable in large-scale surveys. The shorter CES-D8 reduced participant burden and improved response rates. Empirical studies have shown no significant differences between the two scales in their analytical results, supporting the use of the abbreviated version. To ensure comparability between the CES-D8 and CES-D20, the scores from the two forms were equated using the equipercentile equating method, allowing data from both scales to be integrated seamlessly (Wu and Qian, 2024; Lander and Yu, 2025). Additionally, while the original CES-D assigns a score range of 0–3 for each item, the CFPS dataset uses a range of 1–4. Therefore, the total score range for CES-D in CFPS differs from other studies. The CFPS survey clarified that for CES-D 20, the original scale has a score range of 0–60, while the CFPS dataset ranges from 20–80. To make the CFPS scores comparable to the original, 20 points should be subtracted from the CFPS score. In this study, veterans’ depression levels are based on the CES-D 20 results from the 2022 CFPS survey, and according to the guidance of the CFPS survey organization, the original scores were reduced by 20.

Occupational factors. Studies have shown that job satisfaction is defined as an individual’s perception of how well their job outcomes align with their desires, as well as their judgment of how well their job meets their needs. It is regarded as an aspect of wellbeing in a specific domain, closely related to mental health (Oshagbemi, 1999; Magee, 2013). Occupational prestige, which refers to the social status and respect associated with various jobs, can significantly impact multiple aspects of an individual’s mental health, including the risk of depression (Ganzeboom et al., 1992; Thai and Nguyen, 2024). Job stability plays a crucial role in shaping mental health outcomes, such as depression, and can be assessed by the frequency of job changes within a given period (Adams et al., 2013). Job decision authority, which refers to an individual’s ability to make decisions about their job and influence work group or company policies or both, is meaningfully linked to depression (Mausner-Dorsch and Eaton, 2000). In occupational health and organizational research, job control/autonomy refers to the degree of discretion and influence employees have over their work, including decision authority and how tasks are carried out (Hackman and Oldham, 1975). Employees in managerial roles typically report higher levels of job control and discretion than non-managerial employees (Inanc et al., 2013). Moreover, supervisory status, defined as whether an individual supervises others and/or the number of subordinates, is widely used as an indicator of workplace authority (Blommaert et al., 2020). While not a comprehensive measure of job control, “having subordinates” can reasonably approximate the decision-authority dimension in our fsQCA calibration. However, it should be acknowledged that supervisory status, although a reasonable proxy in large-scale surveys, may not capture the full spectrum of job control. For example, it may overlook aspects such as discretion over work pace, task variety, and scheduling flexibility. Receiving lower pay is associated with an increased risk of depression (Theodossiou, 1998). Individuals in informal jobs tend to experience a higher prevalence of major depressive symptoms compared to those in formal employment, with employment status determined by the presence of a formal labor contract (Huynh et al., 2022; Cooke, 2011). The presence of a formal labor contract in this study is based on self-reported responses to the question: Do you have a contract for this job? This data is collected directly from the respondents and reflects their self-reported employment agreement status.

Variables Definitions in this study. Based on the findings of the aforementioned studies, the selected variables from 2022 CFPS, which are relevant to the research objectives, are presented in Table 1.

**Table 1:** Variables’ definitions.

Variables	Variables definition
Depression	Value of CESD-20
Job satisfaction	How satisfied are you with this (current) job?
Occupational prestige	Erikson Goldthorpe Portocarero (EGP) Code
Job stability	Generate number of new jobs during recalling period
Job decision authority	Do you have any direct subordinates in the company/work unit/business of this job
Payment for work	How much in total did you make from this job for the last 12 months? (unit: Yuan)
Formal job	Do you sign a contract for this job?

### 2.4 Variable calibration

The anchor points used in this study are presented in Table 2.

**Table 2:** Variables and values for veterans’ occupational status and depression.

Variables	Original values	Reset value	Variable calibration
Depression	Value of CESD-20	–	15 (0), 21 (0.5), 26 (1)
Job satisfaction	1-Very unsatisfied, 2-Somewhat unsatisfied, 3-Fair, 4-Somewhat satisfied, 5-Very satisfied	–	1 (0), 2 (0.25), 3 (0.5), 4 (0.75), 5 (1)
Occupational prestige	1-Higher controllers, 2-Lower controllers, 3-Routine nonmanual, 4-Self-employed with employees, 5-Self-employed without employees, 7-Manual supervisor, 8-Skilled manual, 9-Semi-unskilled manual, 10-Agricultural laborers, 11-Self-employed agricultural workers	11-Higher controllers, 10-Lower controllers, 9-Routine nonmanual, 8-Self-employed with employees, 7-Self-employed without employees, 5-Manual supervisor, 4-Skilled manual, 3-Semi-unskilld manual, 2-Agricultural laborers, 1-Self-employed agricultural workers.	1 (0), 6 (0.5), 11 (1)
Job stability	Generate number of new jobs during recalling period	>0-unstability, 0-stability	0 (1), >0 (0)
Job decision authority	1-Yes (have subordinates), 5-No (have no subordinates)	1-have subordinates, 0-have no subordinates.	1 (1), 0 (0)
Payment for work	How much in total did you make from this job for the last 12 months? (unit: Yuan)	–	5,000 (0.05), 45,000 (0.50), 140,000 (0.95)
Formal job	1-Yes (sign a formal labor contract), 0-No (not sign a formal labor contract)	–	1 (1), 0 (0)

Variable calibration is a critical step in fsQCA. The cutoff points for the CESD-20 severity categories are based on established theoretical standards from previous research. According to Chen et al. (2022), the CESD-20 severity categories are classified as: None/minimal (≤15), Mild (16–20), Moderate (21–25), and Severe (≥26). Based on these cutoff points, this study calibrated the continuous CESD-20 depression score into fuzzy-set membership scores using three qualitative anchors: 15 as the threshold for full non-membership (membership score = 0), 21 as the crossover point or point of maximum ambiguity (membership score = 0.5), and 26 as the threshold for full membership (membership score = 1). In this calibration scheme, higher membership scores indicate a higher degree of membership in the set of individuals with depression. This calibration approach ensures consistency with existing literature and the theoretical classification of CESD-20 severity levels (Kim et al., 2017).

We employed various calibration methods for different variables. For example, the five-point occupational satisfaction scale was calibrated into fuzzy-set membership scores using the method described in fsQCA literature. This approach assigns fuzzy membership anchors at 0, 0.25, 0.5, 0.75, and 1, corresponding to the ordered categorical responses on the Likert scale, as used in recent empirical fsQCA studies (Othman et al., 2010). Regarding occupational prestige, we followed standard fuzzy-set calibration guidelines and calibrated the ordinal occupational prestige scale using three qualitative anchors. Specifically, the lowest occupational prestige category, self-employed agricultural workers, coded as 1, was set as the threshold for full non-membership with a membership score of 0; the midpoint value of 6 was set as the crossover point or point of maximum ambiguity with a membership score of 0.5; and the highest occupational prestige category, higher controllers, coded as 11, was set as the threshold for full membership with a membership score of 1. This calibration approach is consistent with Ragin’s framework for fuzzy sets (Ragin, 2008). For job stability, job decision authority, and formal job status, these variables were treated as crisp-set binary conditions and calibrated accordingly. Following crisp-set calibration principles, binary variables were assigned membership scores of either 0 or 1. Specifically, 0 represents full non-membership (the individual does not meet the condition), while 1 represents full membership (the individual fully meets the condition). This binary treatment is consistent with the standard calibration approach for crisp-set variables in fsQCA, as outlined by Ragin (2009) and applied by Kahwati et al. (2016). Lastly, for income, the calibration points were set as follows: 5,000 (0.05), 45,000 (0.50), and 140,000 (0.95). Veterans often compare their income with that of the general public rather than solely within their own group. Therefore, the calibrated anchor value for their annual income is based on the income level of the general population. For other variables, calibration anchors were derived from the research of Emmenegger et al. (2014) and Rihoux and Ragin (2009). These points were chosen based on several data sources reflecting economic realities. The lower threshold of 5,000 was based on the minimum standard for urban and rural subsistence living reported by the Ministry of Civil Affairs, approximately 5,000 RMB per year in 2022. The middle threshold of 45,000 was derived from the median per capita disposable income for urban residents, which was reported as 45,123 RMB in 2022. The upper threshold of 140,000 was based on the personal income tax threshold, where individuals earning 144,000 RMB per year are taxed at a higher rate of 20%, indicating a high-income bracket. These reference points were used to construct a calibrated income scale for veterans that reflects both policy standards and actual income distribution in China.

## 3 Results

### 3.1 Necessity analysis

Before conducting the combined path analysis of the conditional variables, we performed a univariate necessity test for each condition using consistency and coverage metrics, which is a standard practice in fsQCA research. In fsQCA, a necessary condition must be present for the outcome to occur and cannot be replaced by other conditions (Vis and Dul, 2018). Consistency measures the degree to which the membership in the outcome set is a subset of the condition set, with a higher consistency score indicating a stronger necessary relationship (Schneider and Wagemann, 2010). A condition is considered necessary if its consistency score is ≥0.90, and it shows a non-negligible coverage value, which reflects empirical relevance (Ragin, 2008; Geremew et al., 2024). A consistency threshold of 0.90 is commonly used because it strikes a balance between methodological rigor and empirical complexity. Coverage, while not having a strict threshold, represents the proportion of outcome membership explained by the necessary condition. In our analysis, a consistency value above 0.90, along with substantial coverage, was interpreted as evidence that the condition is a necessary prerequisite for depression outcomes in the fsQCA model. The necessity analysis for each condition variable was performed using fsQCA 3.0 software, as shown in Table 3.

**Table 3:** Analysis of necessity.

Conditions tested	High depression		Low depression	
	Consistency	Coverage	Consistency	Coverage
Job satisfaction	0.704	0.158	0.727	0.902
~Job satisfaction	0.566	0.273	0.322	0.857
Occupational prestige	0.513	0.163	0.518	0.907
~Occupational prestige	0.708	0.210	0.522	0.855
Job stability	0.686	0.157	0.670	0.843
~Job stability	0.314	0.147	0.330	0.853
Job decision authority	0.091	0.075	0.203	0.925
~Job decision authority	0.909	0.171	0.797	0.829
Payment for work	0.638	0.180	0.617	0.914
~Payment for work	0.680	0.243	0.441	0.871
Formal job	0.504	0.120	0.664	0.879
~Formal job	0.496	0.211	0.336	0.789

~ means negated (lack of the causal condition). For example, “~Job satisfaction” represents low capacity which means veterans’ job satisfaction is at a low level; “~Formal job” represents the job is an un-formal job which means veterans have no formal job contracts.

The results indicate that all conditions, except for “Job decision authority”, do not serve as necessary conditions for high depression. Based on the studies by Legewie (2013) and Goicolea et al. (2018), in fsQCA, once necessary conditions are identified, they should be excluded from the Boolean minimization or truth table analysis. This exclusion is important because necessary conditions appear in all configurations that produce the outcome, making their inclusion in the minimization process redundant. Including them would result in ineffective simplifications and hinder the identification of core causal combinations. Therefore, “Job decision authority” will be excluded from the subsequent configuration analyses of high depression levels among veterans. Additionally, none of the conditions are necessary for low depression.

To further assess the robustness of the necessity analysis, we conducted two additional calibration-based robustness tests. First, we recalibrated the antecedent conditions while keeping the outcome variable unchanged. Specifically, three non-dichotomous conditions were recalibrated: job satisfaction, occupational prestige, and payment/income. Job satisfaction was recalibrated from the original five-level membership scores of 0, 0.25, 0.50, 0.75, and 1 to a less extreme linear calibration scheme of 0.05, 0.275, 0.501, 0.725, and 0.95. Occupational prestige was recalibrated by shifting the crossover point from 6 to 6.5 while retaining the original full non-membership and full membership anchors at 1 and 11, respectively. Payment was recalibrated by keeping the full non-membership and full membership anchors unchanged at 5,000 and 140,000, respectively, while increasing the crossover point from 45,000 to 50,000. For all recalibrated fuzzy-set variables, membership scores exactly equal to 0.50 were adjusted to 0.501 to avoid ambiguity at the qualitative crossover point.

The dichotomous conditions, namely job stability, job decision authority, and formal employment, were not recalibrated in the robustness test. This decision is theoretically and methodologically justified because these variables were calibrated as crisp sets. In such cases, a value of 1 indicates full membership in the set, whereas a value of 0 indicates full non-membership. Artificially assigning fractional membership scores to these binary variables would change their substantive meaning rather than test the robustness of calibration decisions. Therefore, only the continuous or ordinal antecedent conditions were subjected to recalibration.

The results based on the recalibrated antecedent conditions were highly consistent with the baseline necessity analysis. For high depression, the absence of job decision authority remained the only condition meeting the conventional necessity threshold, with a consistency score of 0.908947 and a coverage score of 0.171343. None of the recalibrated antecedent conditions reached the 0.90 consistency threshold. Specifically, recalibrated job satisfaction showed a consistency of 0.710240 and a coverage of 0.163209, while its negation showed a consistency of 0.585616 and a coverage of 0.270225. Recalibrated occupational prestige had a consistency of 0.496437 and a coverage of 0.162871, whereas its negation had a consistency of 0.721299 and a coverage of 0.207817. Recalibrated payment/income showed a consistency of 0.607390 and a coverage of 0.172204, while its negation showed a consistency of 0.706677 and a coverage of 0.236212. These values indicate that the recalibration of the antecedent conditions did not alter the substantive conclusion that only the absence of job decision authority constitutes a necessary condition for high depression among veterans.

For low depression, the robustness results also remained stable. After recalibrating the antecedent conditions, none of the conditions reached the 0.90 consistency threshold. The consistency scores of recalibrated job satisfaction, occupational prestige, and payment/income were 0.713433, 0.501793, and 0.585960, respectively, all clearly below the threshold for necessity. Although some conditions showed relatively high coverage values, such as recalibrated payment/income with a coverage of 0.916839, recalibrated occupational prestige with a coverage of 0.908564, and recalibrated job satisfaction with a coverage of 0.904778, their consistency scores were insufficient to support a necessary-condition interpretation. This finding confirms that no single antecedent condition is necessary for low depression.

Second, we examined whether the necessity results were sensitive to the calibration of the outcome variable. In this robustness test, the antecedent conditions were kept unchanged, while the depression outcome was recalibrated. The full non-membership and full membership anchors were retained at 15 and 26, respectively, whereas the crossover point was increased from 21 to 22. This adjustment represents a stricter calibration of the high-depression set without changing the theoretical meanings of the two endpoints. As with the antecedent recalibration, membership scores located exactly at the crossover point were adjusted to 0.501.

The results based on the recalibrated outcome variable again supported the robustness of the baseline necessity findings. For high depression based on the recalibrated outcome, the absence of job decision authority continued to exceed the 0.90 necessity threshold, with a consistency score of 0.912109 and a coverage score of 0.157060. No other condition reached the threshold. The closest values were observed for the absence of occupational prestige, with a consistency of 0.724249 and a coverage of 0.196527; the absence of payment/income, with a consistency of 0.698477 and a coverage of 0.228260; job satisfaction, with a consistency of 0.713212 and a coverage of 0.146499; and job stability, with a consistency of 0.685273 and a coverage of 0.142880. These results indicate that the identification of the absence of job decision authority as a necessary condition for high depression is not dependent on the original calibration of the depression outcome.

For the negation of the recalibrated depression outcome, none of the conditions met the 0.90 consistency threshold. Although several conditions had high coverage values, including job decision authority with a coverage of 0.933870, payment/income with a coverage of 0.926033, occupational prestige with a coverage of 0.920101, and job satisfaction with a coverage of 0.916919, their corresponding consistency scores were only 0.202261, 0.615164, 0.517475, and 0.727443, respectively. Thus, these conditions cannot be regarded as necessary for low depression.

Overall, the robustness tests provide strong support for the stability of the necessity analysis. Across the baseline model, the antecedent recalibration model, and the outcome recalibration model, the absence of job decision authority consistently exceeded the 0.90 consistency threshold for high depression, with consistency scores ranging from 0.908947 to 0.912109. By contrast, no other condition reached the necessity threshold under any specification, and no condition was found to be necessary for low depression. The coverage values for the absence of job decision authority remained modest, ranging from 0.157060 to 0.171343, suggesting that this condition should be interpreted as a stable but empirically delimited necessary condition rather than as a sufficient or broadly explanatory factor. Therefore, the robustness tests confirm that the main necessity conclusion is not an artifact of specific calibration choices: the absence of job decision authority is a necessary condition for high depression among veterans, whereas no single condition is necessary for low depression.

### 3.2 Condition configuration analysis

In analyzing the configurations of sufficient conditions, this study explores the combinations of conditions leading to high depression and the causal configurations associated with low depression, employing a mutual comparative validation between the two. In the fsQCA 3.0 analysis, three solutions are generated at varying levels of complexity: the complex solution, the parsimonious solution, and the intermediate solution. Our primary focus is on the intermediate solution, with a cross-validation approach employed using the parsimonious solution to ensure the robustness and reliability of the findings.

Following Ragin’s (2006) methodology, a consistency threshold of 0.80 was established for the analysis. A score of 1 was assigned to a configuration if its consistency surpassed this threshold, while a score of 0 was assigned otherwise. Moreover, the case frequency threshold was set to 2, ensuring that only configurations observed in at least two cases were considered. The primary objective of this study was to identify the configurations of job-related factors that contribute to both high and low levels of depression among veterans. The specific configurations of antecedent conditions associated with both high and low depression are summarized in Table 4.

**Table 4:** Antecedent configurations of depression (Source: own study).

Conditions tested	High depression	Low depression (LD)									
		LD1	LD2	LD3	LD4	LD5	LD6	LD7	LD8	LD9	LD10
Job satisfaction	No significant configurations		•	●	•	•	⊗		•	•	
Occupational prestige		⊙	●		●		⊙		●		⊙
Job stability				●			⊗	•	⊙		⊙
Job decision authority		⊙	⊙	⊙			⊙	⊙		⊙	⊙
Payment for work					●	●		⊙		●	●
Formal job		●				●		●	●		
Consistency		0.890911	0.906263	0.887640	0.941742	0.951505	0.869586	0.906354	0.986889	0.920232	0.921447
Raw coverage		0.308928	0.323179	0.400077	0.374922	0.373440	0.085457	0.181435	0.079193	0.417627	0.099852
Unique coverage		0.043614	0.004830	0.022141	0.041988	0.021520	0.011955	0.005165	0.006743	0.000000	0.002295
Solution consistency		0.889748									
Solution coverage		0.770312									

● = core causal condition present; • = peripheral causal condition present; ⊗ = core causal condition absent; ⊙ = peripheral causal condition absent; blank spaces indicate “do not care”.

Table 4 shows that, for high-depression outcomes, no significant configurations were identified, indicating that the measured occupational conditions did not form a stable sufficient causal pattern for high depression. This finding was further supported by the robustness checks. Specifically, we attempted to relax the truth-table criteria by setting the consistency threshold at 0.80 and 0.75, respectively, and by using both frequency cutoffs of 1 and 2. However, under these alternative threshold specifications, the fsQCA procedure still did not return valid sufficient configurations for high depression. This suggests that the absence of high-depression configurations is unlikely to be an artifact of overly strict threshold settings. Rather, it indicates that high depression among veterans may be shaped by causal mechanisms beyond the measured occupational participation conditions, such as trauma history, social support, family relationships, previous mental health status, or other unobserved heterogeneity.

For low-depression outcomes, Table 4 shows that 10 causal configurations led to low depression, with acceptable overall solution consistency and coverage (solution consistency = 0.890; solution coverage = 0.770). More specifically, the 10 configurations for low depression were as follows: LD1 “~Occupational prestige * ~Job decision authority * Formal job”; LD2 “Occupational prestige * ~Job decision authority * Job satisfaction”; LD3 “Job stability * ~Job decision authority * Job satisfaction”; LD4 “Occupational prestige * Payment for work * Job satisfaction”; LD5 “Payment for work * Formal job * Job satisfaction”; LD6 “~Occupational prestige * ~Job stability * ~Job decision authority * ~Job satisfaction”; LD7 “Job stability * ~Job decision authority * ~Payment for work * Formal job”; LD8 “Occupational prestige * ~Job stability * Formal job * Job satisfaction”; LD9 “~Job decision authority * Payment for work * Job satisfaction”; and LD10 “~Occupational prestige * ~Job stability * ~Job decision authority * Payment for work”. These results indicate that low depression among veterans is not produced by a single antecedent condition, but by multiple equifinal combinations of occupational and work-related factors.

The robustness checks for low depression further confirmed the substantive stability of the baseline configurational findings. When the consistency threshold was increased while keeping the frequency cutoff at 2, the number of complex configurations decreased from 10 to 7, and the solution coverage decreased from 0.770 to 0.700. However, the solution consistency increased from 0.890 to 0.911, indicating that the remaining configurations were more internally consistent under the stricter criterion. In addition, when the frequency cutoff was reduced from 2 to 1 while maintaining the baseline consistency threshold, the number of complex configurations increased from 10 to 13, while the overall solution consistency and coverage remained highly similar to the baseline model, with solution consistency increasing slightly from 0.890 to 0.892 and solution coverage increasing from 0.770 to 0.790. More importantly, the parsimonious solution under this relaxed frequency threshold was identical to that of the baseline model. These results suggest that the baseline configurations leading to low depression are not artifacts of specific threshold choices.

One of the core principles of QCA is the recognition that causal relationships can exhibit asymmetry (Ragin and Rihoux, 2004). Previous studies have shown that high depression and low depression are distinct constructs, each with different correlates and causes. For instance, studies using fsQCA approaches with Chinese samples have demonstrated that high depression levels are more strongly associated with clusters of psychosocial stressors, whereas factors such as self-esteem serve as protective factors against depression, indicating divergent causal configurations between high and low depression outcomes (Wang et al., 2025). Measures to prevent or mitigate high depression should therefore differ from those aimed at enhancing low depression (Zhao et al., 2023; Keyes, 2002). The present fsQCA results are consistent with this asymmetric logic: stable sufficient configurations were identified for low depression, whereas no robust sufficient configuration was found for high depression, even after relaxing the truth-table thresholds. This asymmetric pattern supports existing evidence regarding the differential roles of depression-protective factors (Luthar, 2015). However, the absence of sufficient configurations for high depression does not imply that occupational factors are irrelevant to depression. Rather, it suggests that the measured occupational participation conditions alone were insufficient to generate consistent high-depression pathways across cases. High depression may depend more strongly on additional individual, family, and psychosocial conditions that were not included in the present model.

Overall, the configurational results can be retained. The robustness analyses indicate that the low-depression configurations are substantively stable, whereas the absence of high-depression configurations remains unchanged even under more relaxed analytical thresholds. Therefore, the main conclusion is that occupational participation factors are more effective in explaining the presence of low depression than in explaining the occurrence of high depression among veterans.

Among the configurations leading to low depression, several pathways showed relatively high raw coverage, indicating that these configurations explained a substantial proportion of low-depression cases. Specifically, LD9, LD3, LD4, LD5, LD2, and LD1 all had raw coverage values above 0.30, and their consistency scores remained at a relatively high level. This suggests that these pathways not only covered a meaningful share of cases but also demonstrated sufficient consistency in explaining low-depression outcomes.

First, LD9 (~Job decision authority * Payment for work * Job satisfaction) had the highest raw coverage, with a raw coverage of 0.417627 and a consistency of 0.920232. This configuration indicates that, even when veterans lacked job decision authority, low depression could still emerge when adequate payment for work and high job satisfaction were present. This finding suggests that the absence of job decision authority does not necessarily lead to unfavorable mental health outcomes. When economic rewards and positive work experiences coexist, they may compensate for the lack of autonomy in job decision-making. In other words, for some veterans, stable economic returns and subjective satisfaction with work may be more directly associated with lower depression than job decision authority itself.

Second, LD3 (Job stability * ~Job decision authority * Job satisfaction) had a raw coverage of 0.400077 and a consistency of 0.887640. This pathway shows that, even in the absence of job decision authority, the combination of job stability and job satisfaction can lead to low depression. Compared with LD9, the compensatory condition in LD3 is not payment for work but job stability. This result suggests that job stability may provide veterans with predictable life arrangements and a sense of security, while job satisfaction further strengthens positive work-related experiences. Together, these two conditions may buffer the negative implications of limited job decision authority. Although the consistency of this pathway is slightly below 0.90, it remains at a relatively high level and has meaningful explanatory value.

Third, LD4 (Occupational prestige * Payment for work * Job satisfaction) had a raw coverage of 0.374922 and a consistency of 0.941742. This pathway indicates that veterans are more likely to experience low depression when occupational prestige, payment for work, and job satisfaction are simultaneously present. This can be understood as a “occupational status–economic reward–subjective satisfaction” pathway. Occupational prestige may enhance social recognition and self-worth, payment for work provides economic security, and job satisfaction reflects a positive subjective evaluation of employment. The joint presence of these three conditions forms a relatively complete occupational protection mechanism. The high consistency score further indicates that this configuration has strong sufficiency in explaining low-depression outcomes.

Fourth, LD5 (Payment for work * Formal job * Job satisfaction) had a raw coverage of 0.373440 and a consistency of 0.951505. This pathway had the highest consistency among the high-coverage configurations, indicating strong reliability as a sufficient pathway to low depression. It suggests that veterans are more likely to report low depression when formal employment, adequate payment for work, and job satisfaction coexist. Formal employment usually implies more standardized labor relations, stronger institutional protection, and clearer occupational identity. Payment for work provides economic security, while job satisfaction reflects positive psychological evaluation. Together, these three conditions constitute an important protective pathway based on institutional employment, economic reward, and subjective wellbeing.

Fifth, LD2 (Occupational prestige * ~Job decision authority * Job satisfaction) had a raw coverage of 0.323179 and a consistency of 0.906263. This configuration shows that, even when job decision authority is absent, the presence of occupational prestige and job satisfaction can still produce low depression. This suggests that occupational prestige and job satisfaction may jointly compensate for insufficient job autonomy. For veterans, work with higher social recognition may help maintain identity, dignity, self-evaluation, and social integration, while job satisfaction further reinforces these positive effects. Therefore, LD2 reflects a “occupational identity–subjective satisfaction” protective mechanism.

Sixth, LD1 (~Occupational prestige * ~Job decision authority * Formal job) had a raw coverage of 0.308928 and a consistency of 0.890911. This pathway is noteworthy because it includes both the absence of occupational prestige and the absence of job decision authority, yet still leads to low depression when formal employment is present. This suggests that, for some veterans, even if the job lacks high prestige and decision-making autonomy, formal employment may still contribute to lower depression. Formal employment may provide psychological protection through institutional security, organizational belonging, stable labor relations, and basic social protection. This pathway also indicates that low depression does not always depend on ideal employment conditions characterized by high prestige and high autonomy. Instead, relatively basic but institutionally stable formal employment may also play an important protective role.

## 4 Discussion

### 4.1 Configuration effect analysis

The configuration analysis indicates that the measured occupational factors did not generate stable sufficient pathways to high depression among veterans in this sample. No significant configurations were identified for high-depression outcomes in the baseline analysis. This result was further supported by the robustness checks: even after relaxing the analytical criteria by setting the consistency threshold at 0.80 and 0.75 and using frequency cutoffs of both 1 and 2, the fsQCA procedure still did not return valid sufficient configurations for high depression. Therefore, the absence of high-depression configurations is unlikely to be an artifact of overly strict threshold settings. Rather, it suggests that high depression among Chinese veterans may be shaped by additional mechanisms beyond the occupational participation conditions included in this study, such as trauma history, family support, social support, prior mental health status, or other forms of unobserved heterogeneity.

This finding is consistent with previous research highlighting substantial heterogeneity in depressive symptoms, with different risk factors linked to different levels or subtypes of depression (Fried et al., 2014; Musliner et al., 2016). It also supports the principle of causal asymmetry in QCA, namely that the causal conditions leading to the presence of an outcome are not necessarily the mirror image of those leading to its absence (Ragin and Rihoux, 2004). Although certain occupational factors have been associated with higher depression levels in other populations (Zhang and Chai, 2020), this pattern was not observed in the present sample of Chinese veterans. Existing studies on Chinese veterans also suggest that poor occupational status may be more closely associated with social dissatisfaction, reduced perceived fairness, and collective action, rather than with high depression levels per se (Diamant and O’Brien, 2015; Hu and Tong, 2021). Overall, these findings underscore the need for contextualized interpretation when examining depression across groups and symptom levels.

In contrast, the configuration analysis identified multiple sufficient pathways leading to low depression. Table 4 reports 10 configurations associated with low depression, with an overall solution consistency of 0.890 and a solution coverage of 0.770. These results indicate that low depression among veterans is not determined by a single occupational factor, but is produced through multiple equifinal combinations of occupational and work-related conditions. The robustness checks further confirmed the substantive stability of these low-depression configurations. When the consistency threshold was increased, the number of complex configurations decreased from 10 to 7, while the solution consistency increased from 0.890 to 0.911. When the frequency cutoff was reduced from 2 to 1, the number of complex configurations increased from 10 to 13, while the solution consistency and coverage remained highly similar to the baseline model. More importantly, the parsimonious solution under the lower frequency threshold was identical to that of the baseline model. These results suggest that the baseline configurations leading to low depression are not artifacts of specific threshold choices. Rather than interpreting the 10 configurations as isolated pathways, the results can be theoretically summarized into three broader mechanisms.

The first mechanism is the job satisfaction–economic reward pathway, represented by LD4, LD5, and LD9. These configurations share the repeated presence of job satisfaction and payment for work. This suggests that when veterans are satisfied with their work and receive adequate economic rewards, low depression can emerge even when other occupational conditions are not ideal. For example, in LD9, the absence of job decision authority coexists with payment for work and job satisfaction, indicating that adequate compensation and positive subjective work experience may compensate for limited job autonomy. Thus, job satisfaction and payment for work jointly constitute an important psychological–economic protective mechanism.

The second mechanism is the occupational status–subjective satisfaction pathway, represented by LD2, LD4, and LD8. These configurations highlight the joint role of occupational prestige and job satisfaction. Occupational prestige may enhance veterans’ social recognition, self-worth, and occupational identity, while job satisfaction reflects a positive subjective evaluation of their work experience. Even when job decision authority or job stability is limited, the combination of occupational prestige and job satisfaction may help reduce depressive symptoms by strengthening identity integration and perceived social value. Therefore, occupational prestige should not be interpreted only as an external status indicator; it may also operate through psychological recognition and occupational identity.

The third mechanism is the formal employment–institutional embeddedness pathway, represented by LD1, LD5, LD7, and LD8. These configurations show that formal employment is a stable and important configurational element associated with low depression. In LD1, formal employment appears despite the absence of occupational prestige and job decision authority. In LD7, formal employment appears together with job stability even when payment for work and job decision authority are absent. In LD5 and LD8, formal employment combines with other favorable conditions such as payment, occupational prestige, or job satisfaction. These findings suggest that formal employment may reduce depression by providing institutional security, organizational belonging, stable labor relations, and predictable role identity. This interpretation is also supported by the robustness analysis, in which formal employment remained a parsimonious explanatory term under the stricter consistency threshold. Therefore, formal employment appears to be one of the most stable occupational elements associated with low depression in this study.

It is also important to interpret the roles of job decision authority and occupational prestige cautiously. The absence of job decision authority appeared in several low-depression configurations, but this should not be understood as evidence that limited autonomy is beneficial to mental health. Rather, the results suggest that the effect of job decision authority is conditional and configuration-dependent. In some pathways, limited decision authority coexisted with low depression when other protective occupational conditions, such as payment for work, job satisfaction, job stability, formal employment, or occupational prestige, were present. This finding is consistent with studies suggesting that higher job decision-making authority may sometimes be accompanied by greater work-related stress in certain contexts (Al-Zoubi et al., 2024). Therefore, job decision authority does not appear to function as a uniform protective factor in the present study; instead, its relevance depends on how it combines with other occupational resources. Similarly, occupational prestige should not be interpreted as an isolated or universally comparable protective factor. Although the use of a standardized occupational-prestige indicator and a common calibration procedure improves comparability across occupations in the CFPS sample, the social and psychological meaning of occupational prestige may still vary across industries, local labor markets, and individual career expectations. In the low-depression configurations, occupational prestige appeared mainly in combination with job satisfaction, payment for work, or formal employment, suggesting that its protective relevance may operate through broader configurations involving recognition, economic reward, and subjective work evaluation. Thus, occupational prestige should be understood as a relative and context-sensitive employment-status indicator rather than as a context-free determinant of depression outcomes.

Taken together, these findings indicate that formal employment, job satisfaction, payment for work, occupational prestige, and job stability constitute key configurational elements associated with low depression among veterans. Their effects should be understood configurationally rather than independently. In other words, low depression is not generated by any single favorable occupational condition, but by different combinations of institutional security, subjective satisfaction, economic reward, occupational recognition, and work stability. Among these elements, formal employment appears to be the most stable explanatory element, as it remained important in both the baseline model and robustness checks. Job satisfaction also emerged as a stable key configurational element, but its role was not independent; rather, it operated mainly in combination with job stability, payment for work, occupational prestige, or formal employment. By contrast, job decision authority should not be interpreted as a core condition for low depression, because neither its presence nor its absence appeared in the parsimonious solutions of the baseline model or robustness checks. Although the absence of job decision authority appeared in several complex configurations leading to low depression, its effect seems to be conditional and may be compensated for by other favorable occupational resources. Notably, this does not contradict the necessity analysis, where the absence of job decision authority was identified as a robust necessary condition for high depression. Instead, it highlights that the role of job decision authority differs between necessity and sufficiency analyses, as well as between high and low depression outcomes.

### 4.2 Policy implications for reducing depression among veterans

The results of this study offer practical implications for improving employment support policies for Chinese veterans. The empirical findings suggest that occupational participation factors are more effective in explaining low depression than in explaining high depression. Therefore, occupational policy should not be understood as a complete substitute for clinical mental-health intervention, especially for veterans with high depression. For those veterans, additional assessment and intervention may be needed regarding trauma exposure, family support, social support, and prior mental health conditions. However, for promoting lower depression and better psychological adaptation after military-to-civilian transition, employment-related policies remain highly relevant.

First, government agencies should strengthen formal employment protection for veterans by improving the institutional quality of labor relations. The configuration results show that formal employment appears in several low-depression pathways, especially LD1, LD5, LD7, and LD8. The robustness analysis also indicates that formal employment remains stable under stricter threshold settings. These findings suggest that formal employment is an important configurational element associated with low depression among veterans. However, its role should be interpreted carefully. Formal employment may have a direct institutional effect by providing veterans with clearer occupational identity, organizational belonging, and predictable career expectations during the transition from military to civilian life. At the same time, formal employment may also serve as a proxy for broader employment advantages, including social insurance coverage, legal labor protection, workplace stability, and access to organizational support. In the Chinese context, values such as stability, security, and clear contractual arrangements are widely emphasized, and formal employment contracts are often viewed as markers of commitment and long-term predictability in work relationships (Du and Vantilborgh, 2020; Chen and Funke, 2009). This orientation is also consistent with the enduring influence of the “iron rice bowl” ideal in shaping labor expectations, including among veterans transitioning from military to civilian employment. Therefore, employment-support policies should not treat formal employment as a merely administrative employment category. Instead, they should ensure that formal employment is accompanied by substantive protections, including standardized labor contracts, legally mandated social security benefits, healthcare and pension coverage, and mechanisms to reduce informal or unstable employment arrangements among veterans.

Second, employment-support systems should place greater emphasis on improving person-job fit and job satisfaction. Job satisfaction appeared repeatedly in high-coverage configurations, including LD2, LD3, LD4, LD5, LD8, and LD9. These findings suggest that job satisfaction is not merely an individual psychological attitude, but a key configurational element that operates together with occupational prestige, payment for work, job stability, and formal employment. Therefore, veteran employment-support programs should integrate systematic vocational inclination assessments before job placement. Such assessments should examine veterans’ occupational preferences, transferable skills, educational background, physical and psychological adaptation needs, and expectations regarding civilian work. Based on these assessments, employment agencies can improve the match between veterans and available jobs, reduce maladaptive job placement, and enhance the likelihood of sustained job satisfaction. In practical terms, this requires not only providing job information, but also offering career counseling, job adaptation guidance, and post-placement follow-up services. Better person-job fit may strengthen the positive psychological meaning of employment and thereby support lower depression among veterans.

Third, employment policies should improve the quality of employment by combining fair economic reward with occupational recognition. This recommendation is directly linked to the satisfaction–economic reward pathway represented by LD4, LD5, and LD9. In LD4, occupational prestige, payment for work, and job satisfaction jointly appear in the configuration associated with low depression. In LD5, payment for work, formal employment, and job satisfaction coexist. In LD9, payment for work and job satisfaction are present even when job decision authority is absent. These configurations suggest that adequate compensation is important, but its protective relevance is strengthened when it is accompanied by subjective job satisfaction and occupational recognition. Therefore, veteran employment policy should move beyond the narrow goal of increasing employment rates and should pay closer attention to employment quality. Policy measures should include promoting fair wage standards, improving transparency in remuneration, recognizing veterans’ military experience as valuable human capital, and creating career-development channels that enhance veterans’ occupational identity and perceived social value. This is especially important because low depression appears to be associated not simply with having a job, but with employment configurations that provide both material security and social-psychological recognition.

Finally, transitional employment-support platforms should be developed for veterans who cannot immediately obtain stable, well-matched, or high-quality employment. This recommendation focuses on veterans facing employment instability, weak job fit, or limited access to high-quality civilian positions. It is particularly relevant to configurations such as LD3 and LD7, where job stability appears as an important condition in combination with other occupational factors. Transitional platforms can function as a bridge between military service and stable civilian employment by providing temporary but standardized work opportunities, vocational training, career counseling, psychological adaptation services, and continuous monitoring of employment stability. Unlike general job-placement services, these platforms should focus on reducing transition-related uncertainty and preventing repeated unstable employment experiences. Occupational health service practices can also be incorporated into this process by including job-stability indicators in workplace mental-health audits and by identifying veterans at risk of occupational insecurity or job dissatisfaction. Overall, the policy implication of this study is that reducing depression among veterans requires more than employment placement alone. It requires the construction of supportive employment configurations that combine institutional protection, person-job fit, fair reward, occupational recognition, and employment stability.

## 5 Limitations

This study examines the relationship between depressive symptoms and occupational factors among military veterans. However, it does not include a comparative analysis of within-group differences in the veteran population. Future research should take a population heterogeneity perspective to conduct such analyses. Specifically, studies should investigate how key demographic variables, such as rank, age, service branch, and gender, moderate the relationship between occupational factors and depressive symptoms. It is important to acknowledge that our ability to draw definitive causal conclusions is limited by the cross-sectional nature of the 2022 CFPS data and by the limitations of the fsQCA approach. Future research should therefore adopt longitudinal or experimental designs to better understand causal relationships. While “having subordinates” was used as a proxy for “job decision authority” in this study, this measure may not fully capture the broader concept of job decision authority or control. Job decision authority is a multifaceted concept that includes task choice, control over work pace, and decision-making authority, which are well-established in OHS research. Future studies should incorporate additional measures of job decision authority, such as employees’ perceived control over their work, decision-making freedom, and task selection. In addition, although occupational prestige was measured using a standardized indicator, this measure may not fully capture industry-specific differences or individual expectations regarding occupational status. This study also identifies a significant gap in the absence of configurations for high depression symptoms among veterans. This null finding should be interpreted cautiously, as it may reflect model-specification or calibration constraints. Future research should identify factors contributing to high levels of depression, examine configurations associated with such outcomes, and test alternative model specifications and calibration schemes. Given that 97.4% of the sample were men, the findings mainly reflect occupational pathways among male veterans and should be generalized to female veterans with caution. Future studies should use larger and more gender-balanced veteran samples to examine whether depression-related configurations differ by gender.

## 6 Conclusion

The present study examined the associations between occupational factors and depression among Chinese veterans. Using fsQCA, we conceptualized occupational influences as configurational rather than additive, emphasizing that depression-related outcomes may emerge from specific combinations of occupational conditions. The findings revealed an asymmetric pattern between low and high depression. Ten configurations were associated with low depression, with acceptable overall solution consistency and coverage, whereas no stable sufficient configuration for high depression was identified in either the baseline model or the robustness checks. This suggests that occupational conditions are more useful for explaining the maintenance of low depression than for explaining the occurrence of high depression in this sample.

The low-depression configurations indicate that formal employment, job satisfaction, payment for work, occupational prestige, and job stability are key configurational elements. These factors should not be interpreted as independent determinants; rather, their relevance depends on how they combine with one another. Formal employment appeared to be especially stable, while job satisfaction, payment for work, occupational prestige, and job stability mainly operated through joint configurations. By contrast, job decision authority appeared to be context-dependent. Its absence occurred in several low-depression configurations, but it should not be interpreted as a core condition for low depression.

These findings have practical implications for veteran employment-support policies. Rather than focusing only on job placement, policy interventions should promote formal labor protection, improve person-job fit, ensure fair compensation, enhance occupational recognition, and support employment stability. However, because this study relies on cross-sectional data, the identified configurations should be interpreted as evidence-informed occupational patterns associated with depression outcomes, not as direct evidence of causal or intervention effects. Future longitudinal research is needed to clarify causal direction and to examine additional non-occupational antecedents of high depression among veterans.

## Data Availability

The data used in this study are available at https://opendata.pku.edu.cn/dataset.xhtml?persistentId=doi:10.18170/DVN/45LCSO. Further inquiries can be directed to the corresponding author.

## References

[ref1] Adams A. E. Bybee D. Tolman R. M. Sullivan C. M. Kennedy A. C. (2013). Does job stability mediate the relationship between intimate partner violence and mental health among low-income women? Am. J. Orthopsychiatry 83, 600–608. doi: 10.1111/ajop.12053, 24164531

[ref2] Al-Zoubi Z. Ahmed A. Ahmad Q. Omar B. Hytham B. I. (2024). The impact of work pressure on decision-making effectiveness among department heads in faculties of educational sciences. PLoS One 19:e0304584. doi: 10.1371/journal.pone.0304584, 39088449 PMC11293678

[ref3] Becker K. Bish A. McCormack M. Abell D. (2023). Reconceptualizing identities: veterans’ perspectives on career transition challenges. Hum. Resour. Dev. Q. 34, 155–176. doi: 10.1002/hrdq.21472

[ref4] Belloni M. Carrino L. Meschi E. (2022). The impact of working conditions on mental health: novel evidence from the UK. Labour Econ. 76:102176. doi: 10.1016/j.labeco.2022.102176

[ref5] Blommaert L. Meuleman R. Leenheer S. Butkeviča A. (2020). The gender gap in job authority: do social network resources matter? Acta Sociol. 63, 381–399. doi: 10.1177/0001699319847504

[ref6] Byers A. L. Covinsky K. E. Barnes D. E. Yaffe K. (2012). Dysthymia and depression increase risk of dementia and mortality among older veterans. Am. J. Geriatr. Psychiatry 20, 664–672. doi: 10.1097/JGP.0b013e31822001c1, 21597358 PMC3229643

[ref7] Byers A. L. Yaffe K. (2014). Depression and dementias among military veterans. Alzheimers Dement. 10, S166–S173. doi: 10.1016/j.jalz.2014.04.007 24924668

[ref8] Carleton R. N. Thibodeau M. A. Teale M. J. N. Welch P. G. Avrams M. P. Robinson T. . (2013). The center for epidemiologic studies depression scale: a review with a theoretical and empirical examination of item content and factor structure. PLoS One 8:e58067. doi: 10.1371/journal.pone.0058067 23469262 PMC3585724

[ref9] Carlo A. D. Sterling R. A. Mao J. Fiorella R. P. Fortney J. C. Unützer J. . (2024). Characteristics of veterans with depression who use the veterans choice program of the veterans health administration. Psychiatr. Serv. 75, 349–356. doi: 10.1176/appi.ps.202100731, 37933135 PMC11152459

[ref10] Chen X. Chu N. M. Sharma Basyal P. Vihokrut W. Crews D. Brennan D. C. . (2022). Depressive symptoms at kidney transplant evaluation and access to the kidney transplant waitlist. Kidney Int. Rep. 7, 1306–1317. doi: 10.1016/j.ekir.2022.03.008, 35694557 PMC9174041

[ref11] Chen Y. F. Funke M. (2009). China’s new labour contract law: no harm to employment? China Econ. Rev. 20, 558–572. doi: 10.1016/j.chieco.2009.03.008

[ref12] Cooke F. L. (2011). Labour market regulations and informal employment in China: to what extent are workers protected? J. Chin. Hum. Resour. Manag. 2, 100–116. doi: 10.1108/20408001111179159

[ref13] de Van Vel S. Levecque K. Bracke P. (2009). Measurement equivalence of the CES-D 8 in the general population in Belgium: a gender perspective. Arch. Public Health 67, 1–15. doi: 10.1186/0778-7367-67-1-15

[ref14] Diamant N. J. O’Brien K. J. (2015). Veterans’ political activism in China. Mod. China 41, 278–312. doi: 10.1177/0097700414533631

[ref15] Du J. Vantilborgh T. (2020). Cultural differences in the content of employees’ psychological contract: a qualitative study comparing Belgium and China. Psychol Belg. 60, 132–151. doi: 10.5334/pb.498, 32523710 PMC7274204

[ref16] Elnitsky C. A. Andresen E. M. Hall C. McGarity S. Kerns R. W. Clark M. E. (2013). Access to the US Department of veterans affairs health system: self-reported barriers to care among returnees of operations enduring freedom and Iraqi freedom. BMC Health Serv. Res. 13:498. doi: 10.1186/1472-6963-13-498, 24289747 PMC3893594

[ref17] Emmenegger P. Schraff D. Walter A. (2014). QCA, the truth table analysis and large -N survey data: The benefits of calibration and the importance of robustness tests. In 2ed International QCA Expert Workshop. Zurich, Switzerland, 1–36.

[ref18] Fiedler N. Ozakinci G. Hallman W. Wartenberg D. Brewer N. T. Barrett D. H. . (2006). Military deployment to the Gulf war as a risk factor for psychiatric illness among US troops. Br. J. Psychiatry 188, 453–459. doi: 10.1192/bjp.188.5.453, 16648532

[ref19] Ford J. D. Trestman R. L. Steinberg K. Tennen H. Allen S. (2004). Prospective association of anxiety, depressive, and addictive disorders with high utilization of primary, specialty, and emergency medical care. Soc. Sci. Med. 58, 2145–2148. doi: 10.1016/j.socscimed.2003.08.017, 15047073

[ref20] Fried E. I. Nesse R. M. Zivin K. Guille C. Sen S. (2014). Depression is more than the sum score of its parts: individual DSM symptoms have different risk factors. Psychol. Med. 44, 2067–2076. doi: 10.1017/S0033291713002900, 24289852 PMC4104249

[ref21] Ganzeboom H. B. G. De Graaf P. M. Treiman D. J. (1992). A standard international socio-economic index of occupational status. Soc. Sci. Res. 21, 1–56. doi: 10.1016/0049-089X(92)90017-B

[ref22] Geremew Y. M. Huang W. J. Hung K. (2024). Fuzzy-set qualitative comparative analysis as a mixed-method and analysis technique: a comprehensive systematic review. J. Travel Res. 63, 3–26. doi: 10.1177/00472875231168619

[ref23] Ghai D. (2003). Decent work: concept and indicators. Int. Labour Rev. 142:113. doi: 10.1111/j.1564-913x.2003.tb00256.x

[ref24] Goetsch D. L. Ozon G. (2019). Occupational Safety and Health for Technologists, Engineers, and Managers. New York: Pearson.

[ref25] Goicolea I. Hultstrand Ahlin C. Waenerlund A. K. Marchal B. Christianson M. Wiklund M. . (2018). Accessibility and factors associated with utilization of mental health services in youth health centers: a qualitative comparative analysis in northern Sweden. Int J Ment Health Syst. 12:69. doi: 10.1186/s13033-018-0249-4, 30459827 PMC6234690

[ref26] Goldstein K. E. Pietrzak R. H. Challman K. N. Chu K. W. Beck K. D. Brenner L. A. . (2025). Multi-modal risk factors differentiate suicide attempters from ideators in military veterans with major depressive disorder. J. Affect. Disord. 369, 588–598. doi: 10.1016/j.jad.2024.09.149, 39341292 PMC12265924

[ref27] Hackman J. R. Oldham G. R. (1975). Development of the job diagnostic survey. J. Appl. Psychol. 60:159. doi: 10.1037/h0076546

[ref28] Hu J. Tong W. (2021). Emotional mobilization of Chinese veterans: collective activism, flexible governance and dispute resolution. J. Contemp. China 30, 451–464. doi: 10.1080/10670564.2020.1827356

[ref29] Huynh T. B. Oddo V. M. Trejo B. Moore K. Quistberg D. A. Kim J. J. . (2022). Association between informal employment and depressive symptoms in 11 cities in Latin America. SSM Popul. Health 18:101101. doi: 10.1016/j.ssmph.2022.101101, 35698484 PMC9187523

[ref30] Inanc H. Felstead A. Gallie D. Green F.. (2013). Job Control in Britain: First Findings From the Skills and Employment Survey 2012. London: Centre for Learning and Life Chances in Knowledge Economies and Societies, Institute of Education. Available online at: https://orca.cardiff.ac.uk/id/eprint/67985

[ref31] Kahwati L. Jacobs S. Kane H. Lewis M. Viswanathan M. Golin C. E. (2016). Using qualitative comparative analysis in a systematic review of a complex intervention. Syst. Rev. 5:82. doi: 10.1186/s13643-016-0256-y, 27209206 PMC4875617

[ref32] Karasek R. A. Jr. (1979). Job demands, job decision latitude, and mental strain: implications for job redesign. Admin. Sci. Q. 24, 285–308. doi: 10.2307/2392498

[ref33] Keyes C. L. M. (2002). The mental health continuum: from languishing to flourishing in life. J. Health Soc. Behav. 43, 207–222. doi: 10.2307/3090197, 12096700

[ref34] Kim J. Chung H. Askew R. L. Park R. Jones S. M. W. Cook K. F. . (2017). Translating CESD-20 and PHQ-9 scores to PROMIS depression. Assessment 24, 300–307. doi: 10.1177/1073191115607042, 26423348

[ref35] Lander D. Yu J. (2025). Lockdown, Family Conflict, and Adolescent mental Health in China. Available online at: david-lander.com/research/adolescent-mh-china.pdf

[ref36] Legewie N. (2013). An introduction to applied data analysis with qualitative comparative analysis. Qual Soc Res. 14. doi: 10.17169/fqs-14.3.1961

[ref37] Li Y. Chen Y. Ma B. Chen J. L. Zhong J. Jiang Y. . (2024). Configurations associated with the efficiency of the ophthalmology departments in public hospitals of central South China. PLoS One 19:e0315218. doi: 10.1371/journal.pone.0315218, 39729445 PMC11676925

[ref38] Li Y. Y. Liao X. Y. Zhang X. L. Xiong K. Sun S. J. Miao K. . (2022). Characteristics of depressive symptoms and related factors in Chinese veterans. J Army Med Univ. 44, 1911–1922. doi: 10.16016/j.2097-0927.202202100

[ref39] Luthar S. S. (2015). “Resilience in development: a synthesis of research across five decades,” in Developmental Psychopathology, ed. Cicchetti D.. 3rd ed (Hoboken (NJ): Wiley), 739–795.

[ref40] Magee W. (2013). Anxiety, demoralization, and the gender difference in job satisfaction. Sex Roles 69, 308–322. doi: 10.1007/s11199-013-0297-9

[ref41] Mausner-Dorsch H. Eaton W. W. (2000). Psychosocial work environment and depression: epidemiologic assessment of the demand-control model. Am. J. Public Health 90, 1765–1770. doi: 10.2105/ajph.90.11.1765, 11076247 PMC1446399

[ref42] Missinne S. Vandeviver C. Van de Velde S. Bracke P. (2014). Measurement equivalence of the CES-D 8 depression-scale among the ageing population in eleven European countries. Soc. Sci. Res. 46, 38–47. doi: 10.1016/j.ssresearch.2014.02.006, 24767588

[ref43] Musliner K. L. Munk-Olsen T. Eaton W. W. Zandi P. P. (2016). Heterogeneity in long-term trajectories of depressive symptoms: patterns, predictors and outcomes. J. Affect. Disord. 192, 199–211. doi: 10.1016/j.jad.2015.12.030, 26745437 PMC4761648

[ref44] Oshagbemi T. (1999). Overall job satisfaction: how good are single versus multiple-item measures? J. Manage. Psychol. 14, 388–403. doi: 10.1108/02683949910277148

[ref45] Othman M. Ku-Mahamud K. R. Yahaya M. F. (2010). “Evaluating weapons system using fuzzy approximate reasoning,” in The International Conference on Mathematics and Engineering Physics (ICMEP-5), (Cairo: Military Technical College), 1–11.

[ref46] Pandey B. R. (2025). Rethinking occupational health and safety principles—a systems perspective. J. R. Soc. N. Z. 55, 1362–1383. doi: 10.1080/03036758.2024.2333555, 40756835 PMC12315172

[ref47] Ragin C. C. (2006). Set relations in social research: evaluating their consistency and coverage. Polit. Anal. 14, 291–310. doi: 10.1093/pan/mpj019

[ref48] Ragin C. C. (2008). Redesigning social Inquiry: Fuzzy Sets and Beyond. Chicago (IL): University of Chicago Press.

[ref49] Ragin C. C. (2009). “Qualitative comparative analysis using fuzzy sets (fsQCA),” in Configurational Comparative Methods: Qualitative Comparative Analysis (QCA) and Related Techniques, eds. Rihoux B. Ragin C. C. (Thousand Oaks (CA): Sage), 87–121.

[ref50] Ragin C. C. Rihoux B. (2004). Qualitative comparative analysis (QCA): state of the art and prospects. Qual Methods 2, 3–13.

[ref51] Rantanen J. Muchiri F. Lehtinen S. (2020). Decent work, ILO’S response to the globalization of working life: basic concepts and global implementation with special reference to occupational health. Int. J. Environ. Res. Public Health 17:3351. doi: 10.3390/ijerph17103351, 32408597 PMC7277660

[ref52] Rihoux B. Ragin C. C. (2009). Configurational Comparative Methods: Qualitative Comparative Analysis (QCA) and Related Techniques. Thousand Oaks (CA): Sage.

[ref53] Romaniuk M. Fisher G. Kidd C. Batterham P. J. (2020). Assessing psychological adjustment and cultural reintegration after military service: development and psychometric evaluation of the post-separation military-civilian adjustment and reintegration measure (M-CARM). BMC Psychiatry 20:531. doi: 10.1186/s12888-020-02936-y, 33167907 PMC7654614

[ref54] Romaniuk M. Kidd C. (2018). The psychological adjustment experience of reintegration following discharge from military service: a systemic review. J. Mil. Veterans Health 26, 60–73. doi: 10.3316/informit.850047539340143

[ref55] Sayer N. A. Carlson K. F. Frazier P. A. (2014). Reintegration challenges in U.S. service members and veterans following combat deployment. Soc. Issues Policy Rev. 8, 33–73. doi: 10.1111/sipr.12001

[ref56] Sayer N. A. Frazier P. Orazem R. J. Murdoch M. Gravely A. Carlson K. F. . (2011). Military to civilian questionnaire: a measure of postdeployment community reintegration difficulty among veterans using Department of Veterans Affairs medical care. J. Trauma. Stress. 24, 660–670. doi: 10.1002/jts.20706, 22162082

[ref57] Schneider C. Q. Wagemann C. (2010). Standards of good practice in qualitative comparative analysis (QCA) and fuzzy-sets. Comp. Sociol. 9, 397–418. doi: 10.1163/156913210x12493538729793

[ref58] Shah R. V. Blais R. K. (2025). Negative social exchanges are associated with more severe depressive symptoms above and beyond the effects of positive social exchanges in male US service members and veterans. Mil. Med. 190, e1367–e1372. doi: 10.1093/milmed/usae507, 39520547

[ref59] Shi J. (2025). How population ageing drives up China’s military spending: a securitization framework analysis. China Inf. 39, 3–29. doi: 10.1177/0920203X241300395

[ref60] Siegrist J. (1996). Adverse health effects of high-effort/low-reward conditions. J. Occup. Health Psychol. 1, 27–41. doi: 10.1037/1076-8998.1.1.27 9547031

[ref61] Stewart W. F. Ricci J. A. Chee E. Hahn S. R. Morganstein D. (2003). Cost of lost productive work time among US workers with depression. JAMA 289, 3135–3144. doi: 10.1001/jama.289.23.3135, 12813119

[ref62] Tassew W. C. Nigate G. K. Assefa G. W. Zeleke A. M. Ferede Y. A. (2024). Systematic review and meta-analysis on the prevalence and associated factors of depression among hypertensive patients in Ethiopia. PLoS One 19:e0304043. doi: 10.1371/journal.pone.0304043, 38917087 PMC11198805

[ref63] Thai B. T. H. Nguyen T. N. T. (2024). The mediating role of self-stigma of seeking help in the relationship between perceived occupational prestige and mental health: a cross-sectional study on medical doctors. J. Appl. Soc. Sci. 18, 331–345. doi: 10.1177/19367244241264249

[ref64] Theodossiou I. (1998). The effects of low-pay and unemployment on psychological well-being: a logistic regression approach. J. Health Econ. 17, 85–104. doi: 10.1016/S0167-6296(97)00018-0, 10176317

[ref65] Tran T. V. Canfield J. Chan K. (2016). The association between unemployment status and physical health among veterans and civilians in the United States. Soc. Work Health Care 55, 720–731. doi: 10.1080/00981389.2016.1191582, 27348549

[ref66] Tran A. V. Van Tran H. Luong T. T. Nguyen T. N. (2025). Exploring the relationship between stress, burnout, and occupational accidents in civil construction. Emerging Science Journal, 9, 2110–2117. doi: 10.28991/ESJ-2025-09-04-019

[ref67] Tsai C. F. Ouyang W. C. Chen L. K. Lan C. F. Hwang S. J. Yang C. H. . (2009). Depression is the strongest independent risk factor for poor social engagement among Chinese elderly veteran assisted-living residents. J. Chin. Med. Assoc. 72, 478–483. doi: 10.1016/S1726-4901(09)70411-3, 19762316

[ref68] Tuan T. A. Loan D. T. T. (2025). Predicting stress during sleep from biosensor data: an optimized machine learning framework. J. Hum. Earth Future 6, 640–653. doi: 10.28991/HEF-2025-06-03-010

[ref69] Vis B. (2012). The comparative advantages of fsQCA and regression analysis for moderately large-N analyses. Sociol. Methods Res. 41, 168–198. doi: 10.1177/0049124112442142

[ref70] Vis B. Dul J. (2018). Analyzing relationships of necessity not just in kind but also in degree: complementing fsQCA with NCA. Sociol. Methods Res. 47, 872–899. doi: 10.1177/0049124115626179, 30443090 PMC6195096

[ref71] Wang Z. Zhao J. Wang H. Song D. Feng D. Yu M. . (2025). The influencing factors of depression among Chinese male nursing students: application of decision tree and fsQCA. BMC Nurs. 24:877. doi: 10.1186/s12912-025-03407-z, 40629301 PMC12236039

[ref72] Wu Q. Qian H. (2024). Comparing long-versus short-forms of depression scales in an omnibus longitudinal survey. J. Off. Stat. 40, 457–471. doi: 10.1177/0282423X241249104

[ref73] Zhang N. Chai D. (2020). Objective work-related factors, job satisfaction and depression: an empirical study among internal migrants in China. Healthcare (Basel) 8:163. doi: 10.3390/healthcare8020163, 32527070 PMC7348866

[ref74] Zhao C. Guo J. (2022). Are veterans happy? Long-term military service and the life satisfaction of elderly individuals in China. J. Happiness Stud. 23, 477–508. doi: 10.1007/s10902-021-00410-4

[ref75] Zhao Y. Yang L. Sahakian B. J. Langley C. Zhang W. Kuo K. . (2023). The brain structure, immunometabolic and genetic mechanisms underlying the association between lifestyle and depression. Nat. Ment. Health 1, 736–750. doi: 10.1038/s44220-023-00120-1

